# Blockchain Based Solutions to Mitigate Distributed Denial of Service (DDoS) Attacks in the Internet of Things (IoT): A Survey

**DOI:** 10.3390/s22031094

**Published:** 2022-01-31

**Authors:** Zawar Shah, Imdad Ullah, Huiling Li, Andrew Levula, Khawar Khurshid

**Affiliations:** 1 Department of Information Technology, Sydney International School of Technology and Commerce, Sydney, NSW 2000, Australia; andrew.l@sistc.nsw.edu.au; 2 College of Computer Engineering and Sciences, Prince Sattam Bin Abdulaziz University, Al-Kharj 11942, Saudi Arabia; i.ullah@psau.edu.sa; 3 School of Information Technology, Whitireia Community Polytechnic, Auckland 1010, New Zealand; huiling.li@whiireia.ac.nz; 4 School of Electrical Engineering and Computer Science (SEECS), National University of Sciences and Technology (NUST), Islamabad 44000, Pakistan; khawar.khurshid@seecs.edu.pk

**Keywords:** blockchain, distributed denial of service (DDoS) attacks, internet of things (IoT), mitigation of DDoS attacks

## Abstract

Internet of Things (IoT) devices are widely used in many industries including smart cities, smart agriculture, smart medical, smart logistics, etc. However, Distributed Denial of Service (DDoS) attacks pose a serious threat to the security of IoT. Attackers can easily exploit the vulnerabilities of IoT devices and control them as part of botnets to launch DDoS attacks. This is because IoT devices are resource-constrained with limited memory and computing resources. As an emerging technology, Blockchain has the potential to solve the security issues in IoT. Therefore, it is important to analyse various Blockchain-based solutions to mitigate DDoS attacks in IoT. In this survey, a detailed survey of various Blockchain-based solutions to mitigate DDoS attacks in IoT is carried out. First, we discuss how the IoT networks are vulnerable to DDoS attacks, its impact over IoT networks and associated services, the use of Blockchain as a potential technology to address DDoS attacks, in addition to challenges of Blockchain implementation in IoT. We then discuss various existing Blockchain-based solutions to mitigate the DDoS attacks in the IoT environment. Then, we classify existing Blockchain-based solutions into four categories i.e., Distributed Architecture-based solutions, Access Management-based solutions, Traffic Control-based solutions and the Ethereum Platform-based solutions. All the solutions are critically evaluated in terms of their working principles, the DDoS defense mechanism (i.e., prevention, detection, reaction), strengths and weaknesses. Finally, we discuss future research directions that can be explored to design and develop better Blockchain-based solutions to mitigate DDoS attacks in IoT.

## 1. Introduction

With the rapid development of IoT technology, more and more fields have been involved in this industry, such as smart homes, smart cities, smart transportation, intelligent logistics management, etc. [1]. It is predicted that in 2030 the number of IoT devices used around the world will be close to 125 billion devices [2]. However, there are still issues present in some areas related to IoT including security, privacy, identity management, etc. [3]. Among security issues, Distributed Denial of Service (DDoS) attacks pose a serious threat [4]. Since most IoT devices are resource-constrained with limited memory and computing resources, security protection is lacking in these devices [5]. Attackers exploit the vulnerabilities present in IoT devices and control them as part of botnets to launch DDoS attacks. For example, in 2016, a well-known DDoS attack on DynDNS, a provider of a dynamic Domain Name System (DNS), forced many web services to stop, including Github and Twitter [6]. In addition, not only were the victims unable to provide services but the owners of IoT devices also spent a lot of money on the used bandwidth and power [7].

To detect and mitigate DDoS attacks in IoT, various solutions [8,9,10] have been proposed. In [8], an algorithm to detect and mitigate DDoS attacks on the Constrained Application Protocol (CoAP) is presented. In [9], an algorithm based on the framework of Software Defined Networking (SDN) in IoT is proposed, which uses the rate of grouped messages at the boundary to determine whether DDoS attacks have occurred. Similarly, a real-time DDoS attack detection based on the service information of the network time synchronisation is presented in [10]. However, all the mitigation and detection solutions presented in [8,9,10,11,12] are based on a centralised architecture, which presents a single point of failure. Blockchain is considered as a possible solution to secure IoT as it provides a distributed structure [5,7,13].

With a distributed structure, Blockchain is considered a possible solution and has an important role in mitigating DDoS attacks in IoT. The concept of the Blockchain originated from Bitcoin. It does not rely on a third party to store or verify data but through distributed nodes. Blockchain is a decentralised data structure that is composed of a sequence of blocks. Blockchain is used in many fields, including smart contracts, e-commerce, IoT, etc. [13]. With characteristics of decentralisation, anonymity, persistency and auditability [13,14,15], Blockchain technology has recently been used to prevent DDoS attacks in IoT.

Hence, it is important to carry out a holistic approach and conduct a comprehensive survey about DDoS mitigation in IoT using Blockchain technology. To the best of our knowledge, this is the first survey that discusses advancements in mitigating the DDoS attacks in IoT environments that use the Blockchain as the base technology. We note that there are few recent surveys e.g., [16,17,18,19] that broadly focus on DDoS mitigation using Blockchain with no specific focus on IoT domain. Therefore, in this work, we carry out a detailed survey of various Blockchain-based solutions proposed in the current literature to mitigate the DDoS attacks in IoT. We classify the various Blockchain-based solutions into four categories i.e., Distributed Architecture-based solutions, Access Management based solutions, Traffic Control based solutions and Ethereum Platform based solutions. We discuss working details of all the four types of solutions and then critically evaluate each of them by discussing their strengths and weaknesses. Future research directions are also discussed that outline promising investigation areas. We note that few surveys [20,21,22] have been carried out which discuss how Blockchain can improve security in IoT. However, to the best of our knowledge, no survey in the existing literature classifies and critically evaluates various Blockchain based solutions to mitigate the DDoS attacks in IoT.

The main contributions of this study are: (1) To classify various Blockchain-based solutions presented in the current literature to mitigate DDoS attacks in IoT. (2) To discuss the working principles of Blockchain-based solutions proposed in the existing literature for mitigating DDoS attacks in IoT. (3) To critically analyse the various categories of Blockchain based solutions that mitigate DDoS attacks in the IoT. (4) To propose future research directions that can be explored further to design and develop new Blockchain-based solutions for mitigating DDoS attacks in the IoT.

The rest of this paper is organised as follows: In Section 2, background information related to DDoS attacks and Blockchain is provided. In Section 3 and Section 4, respectively, the literature review of various related surveys and the literature review methodology are presented. In Section 5, four types of Blockchain-based solutions are discussed and critically evaluated. Future research directions are proposed in Section 6. Finally, the paper is concluded in Section 7.

## 2. Background

In this section, the background information related to DDoS attacks and Blockchain is discussed.

### 2.1. Distributed Denial-of-Service (DDoS) Attacks

DDoS attacks aim to consume bandwidth or resources and prevent legitimate users from reaching the services [21,23,24]. Various researchers [21,24] discussed DDoS attacks on the three layers of the IoT architecture. For the perception layer, there are jamming, kill command attacks and de-synchronising attacks to avoid reading data from RFID [21]. In the network layer, the Layer-3 attacks aim to exhaust the victim’s resources with different methods, such as flooding attacks, reflection-based flooding attacks, protocol exploitation flooding attacks and amplification-based flooding attacks [24]. The application-level DDoS attacks (Layer-7 attack) are considered more complicated than layer-3 attacks and are harder to be detected by filters. Applications with the potential to be attacked include DNS, HyperText Transfer Protocol (HTTP), Voice over Internet Protocol (VoIP), etc. [21].

**Figure 1:**
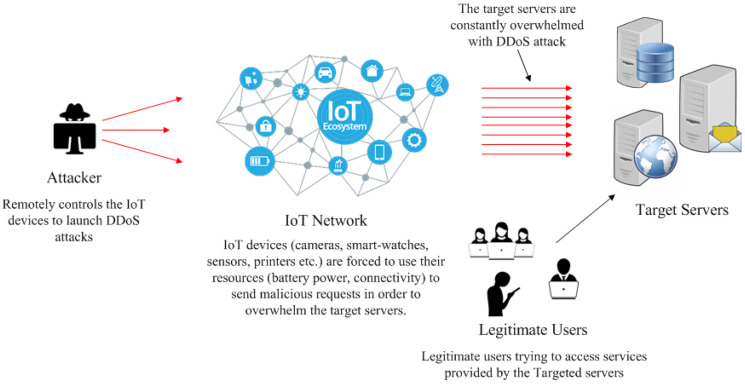
A DDoS attack scenario in IoT networks as a Botnet to target legitimate servers.

The increasing popularity and the ubiquity of IoT, since they are constantly connected to the Internet with negligible security configurations, have made these networks a platform which is strongly exposed to cyberattacks. As a result, these networks have become the new weakest link in the security chain of modern computer networks. Hence, due to their distributed nature, pervasiveness and immense vulnerability, the IoT devices have attracted many bad actors, in particular, those organising for massive DDoS attacks. A DDoS attack scenario within the IoT networks is shown in Figure 1. Such a monstrous attack was done against the KrebsOnSecurity (In-depth security news and investigation: https://krebsonsecurity.com/ accessed on 18 November 2021) in 2016 [25] that knocked out the site for four days and was executed through a network of hacked IoT devices, such as security cameras, video recorders, etc., hit with 620 Gbps of traffic (KrebsOnSecurity Hit With Record DDoS: https://krebsonsecurity.com/2016/09/krebsonsecurity-hit-with-record-ddos/ accessed on 18 November 2021). An even bigger DDoS attack at about the same time was executed using Mirai malware that reached 1.1 Tbps through a collection of hacked Internet-connected cameras and digital video recorders [26].

Although there are slight differences for various strategies that mitigate DDoS attacks, the general defense mechanism contains three parts—prevention, detection and reaction (which is a response to mitigate the attack) [4]. In prevention technologies, filters are commonly used to stop malicious packets. Detection technologies are used to identify attacks and response technologies are used to stop DDoS attacks when they are detected [24].

### 2.2. Blockchain

**Figure 2:**
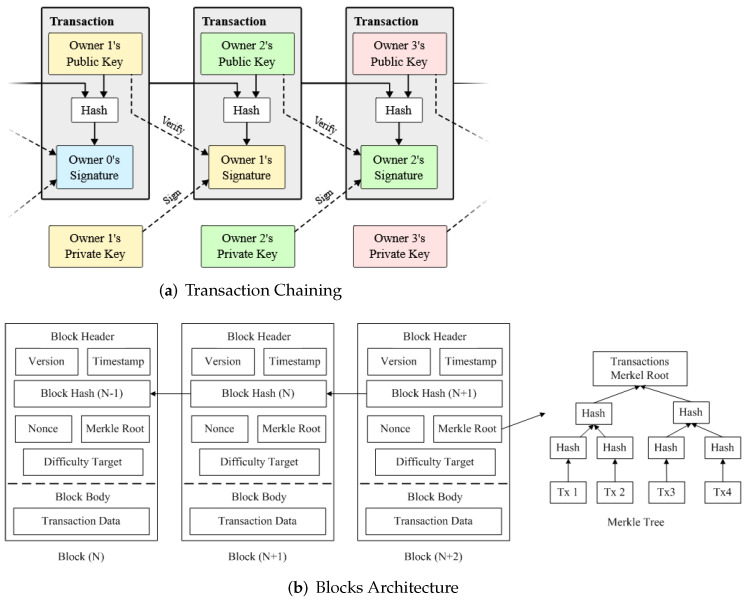
The Chain of Transactions within Blockchain [29], (**a**). the architecture of Blockchain (**b**), and a sample Merkle Tree is also shown.

Blockchain is based on a distributed ledger of transactions (i.e., the communication that takes place between two nodes), composed of a sequence of blocks [27], shared across the participating entities, and provides auditable transactions [28], where the transactions are verified using cryptographic proof [20] by participating entities within the operating network. Blockchain was first proposed by Satoshi Nakamoto, which is also the underlying technology behind Bitcoin [29]. Blockchain has been shown to possess a number of salient features i.e., security, immutability, and hence, could be a useful technology to address various challenges posed by the conventional security systems [30], such as centralised systems with bottleneck and a single point of failure, lack of privacy [31], etc. Contrary to the central trust broker, e.g., Certificate Authority (CA), a node verifies the transactions by validating the signature of the transaction generator against their *Public Key* (PK), which achieves a trustless consensus [32]. In addition, the *Smart Contracts* provide a secure and reliable capability to record and manage interactions for the participating devices [13]. We note that enormous smart-contract-based platforms are emerging that could achieve enhanced functionalities and be used in many application areas, such as IoT [33] and banking services [34]. The Blockchain structure is shown in Figure 2a.

The architecture of a Blockchain is shown in Figure 2b. Each block contains a block header, which includes the timestamp when the current block gets created, nonce, and Merkle root, the Hash value of the previous block (parent block); the first block is called the Genesis block as it has no parent block. Merkle tree, presented on the right side of Figure 2b, is the Hash values of all transactions in a specific block and provides efficient and secure verification of transactions. To form a block, a node periodically collects multiple transactions from the pool of pending transactions, appends it to the local copy of the Blockchain after validating using its PK, and broadcasts to the network [35]; this process is called Mining (Bitcoin mining the hard way: the algorithms, protocols and bytes: https://www.righto.com/2014/02/bitcoin-mining-hard-way-algorithms.html accessed on 18 November 2021). Each node in the participating network uses a Consensus algorithm [36], such as Proof of Work (PoW) [37] and Proof of Stake (PoS) [38] that involves solving a hard-to-solve and easy-to-verify puzzle, to control the participation of nodes within the Blockchain.

There are few comprehensive systematic analysis that examine, compare and contrast on the consensus algorithms [39,40,41,42,43]. The existing Blockchain consensus algorithms can be divided into Byzantine Agreement algorithms (e.g., PBFT [44] ePBFT [45] HoneyBadgerBFT [46]) and Proof-of-X (PoX) (e.g., PoW [37] PoS [38] and Delegated-proof-of-stake (DPoS) [47]). The PoW is computationally expensive [48]; however, there are other algorithms that do not incentivise extreme amounts of energy consumption, e.g., the PoS [38], Proof of Elapsed Time (PoET) [49], Proof of Luck (PoL) [50] and Proof of Space (PoSp) [51], Delegated Proof of Stake (DPoS) [52]. The Byzantine Fault Tolerance (BFT) is used to reach consensus in order to respond with correct information when some of the nodes fail to respond or malicious users trying to propagate false information to their peers. The BFT is implemented in various Blockchains, including Ripple (https://ripple.com/ accessed on 18 November 2021), Hyperledger Fabric (https://www.hyperledger.org/use/fabric accessed on 18 November 2021) and Zilliqa (https://www.zilliqa.com/ accessed on 18 November 2021).

**Figure 3:**
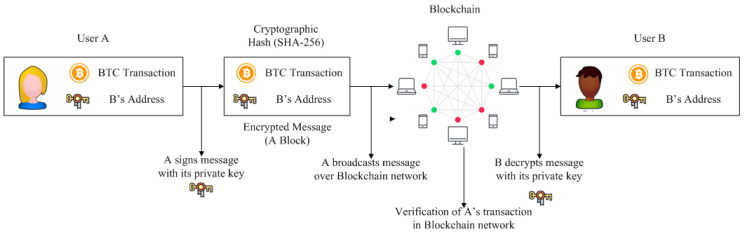
The signing and verification of messages (i.e., transaction data) in a Blockchain network.

An important feature of Blockchain is the signing and verification process of the transactions that are carried out using Digital Signature, which is used to identify users with (owning) a pair of Public and Private Keys. When a user *A* wants to sign a transaction (e.g., transaction data in the form of sending crypto BTC), she first generates a *Hash* value derived from a transaction; then, she signs (i.e., encrypts) this Hash value using her Private Key and sends it to user *B* along with original transaction data. User *B* verifies this transaction by comparing the decrypted Hash (while using *A*’s Public Key) and the Hash value derived from the received data using the same Hash function as *A*’s; an example scenario is shown in Figure 3. The Elliptic Curve Digital Signature Algorithm (ECDSA) is a representative algorithm that is used in the Blockchain [53].

### 2.3. Internet of Things (IoT)

The IoT has become an important part of our daily lives, and it has received immense attention from the research, academia and industry due to its growing need for IoT devices in everyday life [54,55,56,57,58,59]. The IoT combines hardware and software components together [60] as defined by the International Telecommunication Union (ITU) that can concurrently operate various services implemented over TCP/IP and non-TCP/IP protocols [61] according to the definition provided by the Internet Engineering Task Force (IETF). IoT has many applications and deployment scenarios, such as Industrial Internet of Things (IIoT), Internet of Anything (IoA), Internet of Everything (IoE), Social Internet of Things (SIoT), Web of Things (WoT), and Internet of Medical Things (IoMT).

The increased number of connected devices poses several challenges e.g., to effectively handle the enormous amounts of data [62,63], interoperability among various hardware and software platforms [64,65], challenges related to Big data management, privacy and provenance [66], mobility management and handover [67,68,69], and privacy and security challenges [70,71]. We note that there are various efforts from the research community in order to improve the performance and scalability of the IoT netwroks e.g., the use of edge computing to perform resource-intensive tasks [72,73,74], the use of SDN for flexible management and programmability [75,76] including flexible network programmability [77,78] that introduced a new combination of Software-Defined Internet of Things (SDIoT) architecture for an effective management [78,79,80,81,82]; similarly, the Software-Defined Internet of Things and Edge (SDIoT-Edge) frameworks [83,84] were suggested for increased scalability and interoperability [82].

### 2.4. Integration of Blockchain and IoT

The Internet of Things (IoT) refers to the interconnection of smart devices that collect and disseminate data and can be used for making intelligent decisions, which is bringing the capabilities of converting the physical world into a huge information system. The Statista (https://www.statista.com/statistics/802690/worldwide-connected-devices-by-access-technology/ accessed on 18 November 2021) forecast suggests that, by 2030, around 50 billion devices will be in use around the globe. This technology is essentially causing the growth of the ICTs that incorporates various technologies e.g., cloud computing, information modeling, including the Blockchain. An important aspect of IoT in enabling the development of new services in ICT is the data enhancement; however, the lack of security measures makes the IoT vulnerable to security threats [85], such as Distributed Denial-of-Service (DDoS) to exploit the distributed nature of IoT devices [86]. The centralised configuration of IoT networks is a single point of failure, which must be dealt with [87], causing threats to confidentiality and authentication [88]. We note that the Blockchain has various “by-design” potentials that can help with solving major security and privacy issues in IoT by its various capabilities i.e., transparency, immutability, auditability, etc.

In addition, the data aggregated from several sensor nodes are used to make timely decisions e.g., healthcare or other emergency services, which raises the ‘problem of data integrity’. Hence, it is important to protect the data during data collection and dissemination from possible false data injection attacks [87]. Similarly, the correct data availability is another major issue [85] for real-time applications e.g., smart grid, where smart devices’ downtime can result in the loss of real-time system monitoring. Another problem with IoT is ‘non-repudiation’ i.e., the trust among the participating entities in IoT [89] including the central authority; hence, it needs a publicly verifiable audit without the presence of any trusted third party.

### 2.5. Benefits and Challenges of Using Blockchain in IoT

The characteristics of Blockchain include decentralisation, anonymity, persistency and auditability [90]. Recall that these unique features of Blockchain provide a promising solution to the security problems of IoT. It improves the IoT systems in terms of decentralisation, resilience, security and identity management. The current implementation of IoT is composed of a decentralised architecture [91] that poses several challenges such as interoperability, scalability and adaptability surrounding IoT. The Blockchain, hence, can provide a secure platform for IoT networks, thus eliminating decentralised traffic flows and single point of failure. The majority of the participants within the Blockchain must verify the transactions in order to approve them and further add them to the distributed public ledger, which provides publicity and transparency. In addition, there is no single authority for transactions approval or to set specific rules communication or services access for the participants. Hence, there is an improved and huge trust since the majority of the participating IoT network devices must reach an agreement in order to validate transactions.

Despite the benefits of the combination of IoT and Blockchain, there are many challenges, such as scalability, security, algorithms specific for IoT environments, etc. [92]. Since security is the primary concern of this article, the challenges of security are addressed here. Although Blockchain can improve the security of the IoT environment, such as data storage, transaction processing and communication [93], data reliability is still one of the challenges when Blockchain is combined with IoT. Data storage in a Blockchain is permanent. When data of IoT devices are corrupted before they reach the Blockchain, storing such data can cause serious problems in the Blockchain [5]. In addition, IoT devices are likely to be hacked, bugged and controlled by attackers, in addition to the increasing number of attacks against the IoT, Blockchain is itself vulnerable to attacks, such as DDoS attacks, 51% attacks, the Sybil attacks, etc. [14].

## 3. Literature Review

The combination of Blockchain and IoT has attracted a lot of attention and recently many surveys and review articles are published in this area. Some of the surveys [14,92,94] discussed these two technologies in terms of the importance of usage of Blockchain for the security of IoT, challenges and future directions of applying Blockchain in IoT. However, these surveys [14,92,94] did not discuss how Blockchain can be used to detect and mitigate DDoS attacks in IoT. In the surveys conducted by [95,96], the authors reviewed and compared several case studies that utilise Blockchain in IoT to provide security and privacy in different scenarios, such as access control, economic scenarios, smart homes, etc. However, authors in [95,96] did not provide any discussion on mitigating DDoS attacks in IoT. Some researchers analysed the requirements of deploying Blockchain in IoT [5,90,97] and explained how Blockchain is used in IoT but without consideration of DDoS attacks.

The history of DDoS attacks can be traced back to 1998 [4] and, therefore, there is a lot of literature [4,98,99,100] that reviews types of DDoS attacks and various defense mechanisms. However, these surveys are only for traditional networks and do not discuss IoT-based networks. For surveys in the IoT environment, Refs. [15,101] discussed DDoS attacks in three layers of the IoT architecture. However, they did not explain and critically analyse different solutions for mitigating DDoS attacks in IoT. Some researchers [102,103] reviewed the effects of DDoS attacks on IoT, prevention mechanisms and solutions to mitigate DDoS attacks in IoT. In [102], the focus of authors is on the DDoS attacks at the network layer; however, Ref. [103] discussed technologies on an IoT protocol stack to mitigate DDoS attacks, such as IPv6, IEEE 802.15.4, 6LoWPAN, etc. However, Refs. [102,103] did not mention Blockchain-based solutions that mitigate DDoS attacks. Table 1 summarises the results of our literature review.

Our literature review shows that there is no study available in the existing literature that has carried out a survey to analyse the Blockchain-based solutions to mitigate DDoS attacks in IoT. This paper aims to fill in this research gap by categorising and critically evaluating various Blockchain-based solutions to mitigate DDoS attacks in IoT. Another novelty of our work is to propose future work directions that can be explored by other researchers to propose better Blockchain solutions to mitigate DDoS attacks in IoT.

## 4. Research Methodology

The strategy of selecting articles for this study includes search keywords, mentioned databases and the paper selection criteria, which resulted in this survey. Specifically, the keywords such as Internet of Things, IoT, DDoS and Blockchain were utilized to search for the articles from journals and conferences. To summarise, in order to qualify based on our search criteria, a selected article must satisfy the following conditions: (1). the published work must be within the IoT domain, (2). the IoT domain may be generic or application specific (e.g., IIoT), (3). the considered scenario must be of DDoS, IoT and Blockchain, thus excluding all papers that do not either address DDoS attack scenarios or do not use Blockchain as the underlying technology, (4). it must have been published (inclusive) between 2016 and 2021, (5). exclude the review papers written in languages other than English, and (6). the resultant paper should be a Conference or Journal, hence, Books, Newspapers, Dissertations, etc., were excluded.

The research questions that helped provide a systematic and comprehensive survey to analyse the Blockchain-based solutions to mitigate DDoS attacks in the IoT domain are as follows:

- How the attackers exploit IoT networks as a botnet to launch DDoS attacks to target legitimate users, in addition to the severity of the DDoS attacks in IoT domains;
- How the Blockchain may be a candidate technology to mitigate the DDoS attacks;
- What are the current proposals of Blockchain-based solutions used to mitigate the DDoS attacks in the IoT domain, specifically, their working principles, and the DDoS defense mechanism (i.e., prevention, detection, reaction)?
- What are the strengths and weaknesses of the current solutions and the major challenges for designing and implementing comprehensive architectures for implementing Blockchain-based solutions to mitigate DDoS attacks in IoT networks?
- What are the open research areas and challenges for proposing secure Blockchain-based IoT networks (and to suggest the use of other supporting technologies) to mitigate DDoS attacks?

Note that the majority of the search facility of the publishers and databases looks for the combination of keywords e.g., “IoT DDoS Blockchain” in either paper’s title or abstract or the contents, etc. The ACM, instead, provides an advanced search where the keywords can be specifically found in metadata or other parts of a research article including the paper’s title, publication title, paper’s contents, etc., either with ‘AND’ or ‘OR’ operations, to limit or widen the search result. We used various keyword combinations to find the most relevant papers, e.g., “IoT DDoS Blockchain”, “IoT DDoS Mitigation Blockchain” and “IoT DDoS Mitigate Blockchain” that resulted in different number of papers, i.e., with IEEE Xplore, respectively 65, 5, 2. We note that the last two keyword combinations would reduce the number of our search results; hence, we continued our search with the “IoT DDoS Blockchain” keyword combinations to find relevant research articles.

We mainly look for the relevant papers published with various databases i.e., IEEE Xplore, Elsevier, Springer, ScienceDirect and ProQuest based on our criteria. Furthermore, we search for relevant research articles in Google Scholar so as to find articles published with publishers, e.g., MDPI, other than our focused database (we interchangeably use the terms ‘publisher’ and ‘database’). Our search results found 3983 resources that include Conference papers, Journals, Books, Early Access Articles, Magazines, Webpages, Connect, Survey Articles, Discussions, Editorials, Dissertations & Thesis, Newsletter and Reports. Among the search results, we note that there were 1117 Journals and Conference papers and 2866 were other resources e.g., Newsletter, Reports, etc. that were excluded from our repository in the first phase. As an example, based on our search criteria, the IEEE Xplore (https://ieeexplore.ieee.org/Xplore/home.jsp accessed on 18 November 2021) resulted in 65 resources; among them, 32 were Conference papers and seven were Journal articles. The search results from various databases are given in Table 2.

In the second phase, we downloaded the metadata of the searched articles and stored them in our repository in *BibTex* format that includes, author list, title, abstract, keywords, etc. We thoroughly read the relevant metadata i.e., title, abstract and keywords list and stored the (expected list of) relevant papers to be considered in our survey. Finally, to the best of our knowledge and efforts, we found 34 papers that were classified into various Blockchain-based solutions for mitigating DDoS attacks in the IoT domain, 12 survey papers for a comparison with our work, and the remaining 103 were supporting articles that provide generic Blockchain-based solutions for securing IoT networks or contain other supporting materials. Finally, out of 1117 papers, the 1117 − 149 (Total references in this survey are 153, which includes four web-references) = 968 research articles were excluded from our repository that were not classified as Blockchain-based solutions based on our classification. We note that the search facility of the publishers and databases looks for the combination of keywords “IoT DDoS Blockchain” in either paper’s title or abstract or the contents e.g., in ‘Introduction’ section that may discuss about DDoS security attacks in general or the DDoS discussed in the ‘Future Works’ within the paper. Furthermore, we note that there was no duplication of the research articles among individual publishers, since a particular article is published with only one publisher. However, the duplication occurred when we searched for papers in ProQuest or Google Scholar, which we compared with our repository for duplication, deleted them, and then merged them within the repository.

## 5. Blockchain Based Solutions to Mitigate DDoS Attacks in IoT

**Figure 4:**
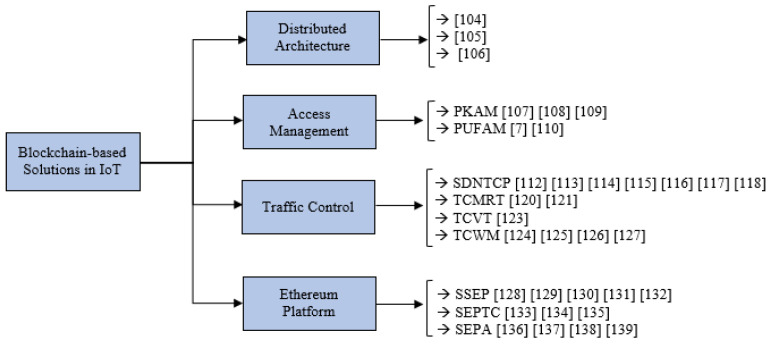
Classification of the blockchain-based solutions to mitigate DDoS attacks in IoT.

In this section, the working principles of various Blockchain-based solutions to mitigate DDoS attacks in IoT are discussed. An important feature of our work is to critically evaluate these solutions and highlight the issues in them. We divide the various Blockchain-based solutions discussed in the current literature into four broad categories, which are: Distributed Architecture-based solutions, Access Management based solutions, Traffic Control based solutions and Ethereum Platform based solutions. This classification is shown in Figure 4.

### 5.1. Distributed Architecture Based Solutions

The distributed architecture-based solutions are proposed in [104,105,106], and they use Blockchain as redundancy data storage, where all the nodes share the ledger with the transactions. These methods take advantage of the distributed structure of Blockchain to mitigate DDoS attacks. This is because it is difficult for an attacker to flood all nodes at once to prevent the system from providing services. Even if some nodes are corrupted, the entire system can continuously provide services [104,105,106].

#### Strengths and Weaknesses of Distributed Architecture Based Solutions

This type of solution utilises the natural feature of Blockchain to prevent DDoS attacks. It uses asymmetric cryptography for identity authentication of IoT devices. However, these solutions [104,105,106] rely solely on the distributed structure of the Blockchain to prevent DDoS attacks. When a DDoS attack occurs, although the whole system can continue to run, the attacked node would be affected. This may further degrade the performance of the whole system. This is because the average latency of processing each transaction increases as the number of transactions processed by each node increases. If the system runs normally under a DDoS attack, the non-working nodes will cause the working nodes to deal with more tasks, which will further reduce the performance of the whole system. This type of solution only prevents DDoS attacks; however, once a hacker takes advantage of the vulnerability of IoT devices to send malicious requests, these systems cannot detect and react to mitigate the attack. The working principle, weaknesses and strengths of these solutions are summarised in Table 3.

### 5.2. Access Management Based Solutions

Solutions in this category take advantage of the fact that DDoS attacks can be mitigated by preventing malicious access. These solutions prevent malicious attackers from accessing the system and allow legitimate users to use the system. Therefore, attackers cannot access the IoT devices to launch DDoS attacks. We divide these solutions into two categories i.e., Public Key based Access Management (PKAM) and Physically Unclonable Function based Access Management (PUF). These two categories are discussed below:

#### 5.2.1. Public Key Based Access Management (PKAM)

PKAM is proposed in [107,108] to prevent DoS attacks in IoT. When a management node receives a transaction request, it determines whether to process or forward the transaction by validating the requester’s public key. If the requester’s public key is not registered or unavailable, the request is forwarded to another management node. Once the request is not accepted by many management nodes after several attempts, the request will be blocked. The public key will be blocked if it sends multiple unsuccessful access requests. Therefore, PKAM mitigates DDoS attacks by verifying access rights and preventing network traffic caused by fraudulent transactions. In addition, to avoid DDoS attacks, the authors in [109] design a Blocikchain-based access control system for IoT devices based on identity-based signature to enable servers to filter and forward access requests.

The strength of this approach is that it is considered IoT access management. In addition, it is a relatively lightweight solution that is suitable for resource-limited IoT devices. Moreover, this method can be used in any IoT industry to control access and protect data security. However, the weaknesses of this approach are (1) if adversaries exploit multiple public keys, they can generate a significant burden on the system, as requests are constantly forwarded and confirmed. This means that the scheme cannot mitigate DDoS attacks in such a scenario. (2) If an attacker can control a device that has been authenticated and make it send malicious requests repeatedly, there is no working mechanism to detect or stop this unwanted traffic.

#### 5.2.2. PUF Based Access Management (PUFAM)

PUFAM provides a unique identity for every IoT device [7,110]. Once a device is registered in the system, the device owner can verify its authenticity anywhere and at anytime. All tampered, fake and cloned devices will be detected, which can prevent devices from becoming part of a botnet [7,110]. In addition, because all the IoT devices are authenticated by Blockchain, the edge network is considered trustworthy and verified. Therefore, the system limits unauthorised access and thus prevents DDoS attacks. Experiments are conducted in [110] to test the system performance under different transaction rates. The results showed that using Blockchain leads to a decrease in performance, such as throughput, latency and CPU usage. The cost increased as the transaction rate is increased. The strength of this PUFAM is that it provides a lightweight access management solution to prevent potential botnet threats. It uses PUF technology to verify the authenticity of the IoT devices. The Blockchain is used to protect data and verify the identity of devices, so as to protect the security and privacy of user data. The weaknesses in this type of solutions is that, once an attacker gets access to the system, there is no mechanism to detect or react to mitigate DDoS attacks.

#### 5.2.3. Strengths and Weaknesses of Access Management Based Solutions

Access management based solutions focus solely on authentication of IoT devices. Their strength is that these solutions considered IoT access management with a relatively lightweight solution. Although to some extent, these solutions can prevent DDoS attacks, there is no comprehensive mechanism to provide defense against DDoS attacks e.g., monitoring transactions or traffic to detect DDoS attacks and then preventing them. Once an attacker finds a way to bypass authentication and launch a DDoS attack, the system has no working mechanism to respond and stop the attack. The working principles, weaknesses and strengths of these solutions are summarised in Table 4.

### 5.3. Traffic Control Based Solutions

The basic working principle of this type of solution is that they utilise traffic control to mitigate DDoS attacks. These solutions monitor transactions to analyse and detect malicious traffic. According to the detection results, the system responds to DDoS attacks with corresponding measures. We divide these solutions into four subtypes i.e., Software Defined Networing based Traffic Control via Blockchain (SDNTCB), Traffic Control based on the Maximum Rate of Transactions (TCMRT), Traffic Control based on Verification of Transactions (TCVT) and Traffic Control based on Whitelisting Mechanism (TCWM). The working principles, strengths and weaknesses of these solutions are explained below:

#### 5.3.1. Software Defined Networking (SDN) Based Traffic Control via Blockchain (SDNTCB)

Recall that the SDN, composed of different layers e.g., the infrastructure layer, control layer and application layer, hence, it may be targeted with DDoS attacks aiming at three different kinds of DDoS attacks i.e., DDoS attacks against the infrastructure, control and application layers [111]. The authors in [112] proposed a blockchain-based decentralised security architecture for IoT networks that consider different SDN controllers as fog nodes. The fog nodes act as processing nodes that detect attacks and where the attack detection model is dynamically updated at the fog nodes. Furthermore, a central cloud server is introduced that manages the attack model from a set of fog nodes. The performance measures, evaluated as of accuracy, detection rate and F-score, show good accuracy with effective DDoS detection.

The key idea of solutions in this category is to utilise SDN based traffic control via Blockchain to mitigate DDoS attacks [113,114,115,116,117,118]. In order to resist DDoS attacks, these solutions combine the SDN and Blockchain to take advantages of both technologies. All SDN controllers in the IoT network are interconnected by the distributed Blockchain network, which enables each IoT device to communicate conveniently and efficiently [113]. With Blockchain technology, security policies and flow rules can be enforced and updated [115]. SDN enabled switches provide dynamic flow management of traffic, which assists in the detection of DDoS attacks [115]. Therefore, solutions under this classification are able to track suspicious traffic and detect DDoS attacks, and thus respond to DDoS attacks [112,113,114,115,116,117,118].

The [113,115] have experimented with preventing DDoS attacks. [113] launched several types of DDoS attacks including TCP/SYN flood, UDP flood and ICMP flood with Stacheldraht tool (which is used to generate traffic). However, Ref. [113] did not give specific experimental results but stated that their solution could accurately detect whether devices are in a botnet through the flow rules on the switches.

Authors in [115] conducted a large-scale experiment with 6000 nodes, which includes three aspects i.e., bandwidth usage, accuracy and CPU utilisation. UDP flooding attack was launched on the SDN switches with different attack rates. The bandwidth of users was analysed, and results show that the bandwidth remained basically unchanged with and without DDoS attacks. As a result, the solution reduced the impact of DDoS attacks without too much resource consumption. In addition, the accuracy of DDoS attacks detection was tested. The results showed that the system can quickly identify DDoS attacks [115]. In addition, the mitigation performance of DDoS attacks is evaluated. The results showed that the proposed solution prevents DDoS attacks in 5 to 7 s and the bandwidth is restored. [114] uses SDN controllers to monitor traffic to add IP addresses into blacklists and whitelists. Similarly, Ref. [116] integrates Blockchain with SDN to manage IoT services.

The strength of these solutions [112,113,114,115,116,117,118] is that they use comprehensive architecture to prevent, detect and mitigate DDoS attacks. The weakness of these solutions [112,113,114,115,116] is that the use of SDN will require additional processing and calculation on controllers, which will lead to a certain delay. However, all these solutions did not calculate that delay. In addition, traffic monitoring follows certain flow rules, and it is possible that unwanted traffic can comply with the established rules but carry out malicious behavior [119]. Moreover, SDN infrastructure is itself vulnerable to many attacks [119] which are not considered by authors in [112,113,114,115,116].

#### 5.3.2. Traffic Control Based on the Maximum Rate of Transactions (TCMRT)

The solutions in this category are characterised by setting a maximum transaction rate to control traffic to mitigate DDoS attacks [120,121]. Nodes in these solutions [120,121] have a threshold for the maximum transaction rate. If the threshold is exceeded, the node manager updates to prevent the node from continuously sending transactions to the target nodes, which can stop DDoS attacks. The strength is that limiting the number of transactions by a threshold can mitigate the impact of a DDoS attack. The weaknesses are that legitimate traffic from users may not get a response in time under an attack. In addition, the dynamic threshold adjustment needs to be realised through network communication. When nodes are active frequently, the whole communication medium will be flooded rapidly. Moreover, the local Blockchain in the system presented by [120] is not distributed, but centralised, which is contrary to Blockchain’s decentralised principle. It will limit its ability and availability [122].

#### 5.3.3. Traffic Control Based on the Verification of Transactions (TCVT)

The solution [123] in this category prevents the nodes from becoming part of a botnet to launch DDoS attacks by validating outgoing transactions. Firstly, the Blockchain will determine whether the device is normal or malicious. Secondly, all outgoing transactions need to be verified through smart contracts. Transactions or traffic that do not conform to the security policy in the smart contract will be rejected. When an adversary gains access and wants to infect the victims, the illegal transactions cannot be sent out and thus the victim will not be attacked [123].

The advantage of this category is that it uses Blockchain without many additional resources. The weakness is that there is no mechanism to protect smart contracts since the solution relies on smart contracts to verify transactions.

#### 5.3.4. Traffic Control Based on the Whitelisting Mechanism (TCWM)

A whitelisting mechanism is used in [124,125,126,127] to prevent DDoS attacks by filtering and eliminating malicious traffic. The whitelisting mechanism controls traffic from data sources, including Internet Protocol (IP) addresses, nodes and users. The whitelisting mechanism usually combines the whitelist and blacklist together to classify users as trusted and untrusted users. There are two types of methods to identify the whitelist—using a tracking manager [124,125] and Machine Learning technology [126,127]. While Refs. [124,125] used tracking managers to identify and record IP addresses in the whitelist, Refs. [126,127] utilised data mining methods to analyse and filter malicious traffic, such as community detection analysis on the mutual contacts graph, neural and mesh networks.

By using this mechanism, it is very quick to verify the access traffic and filter the unwanted traffic. However, these solutions did not consider authentication of IoT devices or how to manage resource limited devices. In addition, similar to flow rules, it is possible that illegal traffic can comply with whitelist validation rules but is actually harmful to the system.

#### 5.3.5. Strengths and Weaknesses of Traffic Control Based Solutions

The strengths of traffic control-based solutions are that they can monitor traffic before or during the processing of each transaction. In addition, experiments are carried out in three solutions in the SDNTCB [113,115] to gauge the feasibility of these solutions to detect and mitigate DDoS attacks. However, all traffic control based solutions did not conduct experiments. Another weakness of the solutions in this category is that monitoring traffic needs additional calculation and processing. For example, solutions in SDNTCB need to calculate delay caused by SDN controllers; Refs. [126,127] in TCWM are required to calculate the likelihood of malicious users based on traffic by using data mining techniques. These additional calculations and processing might lead to a decrease in system performance, such as high latency, increased CPU utilisation, etc. Table 5 summarises the weaknesses and strengths of the solutions in this category.

### 5.4. The Ethereum Platform Based Solutions

The Ethereum platform based solutions take advantage of the mechanism of Ethereum platform to prevent DDoS attacks. This is because each transaction must be paid, which effectively prevents attackers from making too many service requests [128]. The Ethereum Blockchain is a public Blockchain platform that provides an opportunity for developers to write code flexibly on the Blockchain to execute. It uses the concept of “gas” which refers to the fee of conducting transactions or executing smart contracts. Gas is used to allocate resources of the Ethereum virtual machine. If the user does not pay enough gas, the contract will not be fully implemented, and all changes will be rolled back [129]. These types of solutions are further divided into three categories i.e., Solutions Simply based on the Ethereum Platform (SSEP), Solutions based on the Ethereum Platform with Traffic Control (SEPTC) and Solutions based on the Ethereum Platform with Authorization (SEPA). The working principles, strengths and weaknesses of these solutions are explained below:

#### 5.4.1. Solutions Simply Based on the Ethereum Platform (SSEP)

This type of solutions [128,129,130,131,132] uses inherent features of Ethereum platform to prevent DDoS attacks. These solutions [128,129,130,131,132] mentioned that the system is robust to DDoS attacks with the Ethereum platform. This is because the distributed architecture is used and the payment for transactions effectively prevents DDoS attacks by avoiding too many service requests in the Ethereum Blockchain.

The weaknesses of this type of solutions are that these solutions are in the initial stage, and the Ethereum platform is used to quickly implement the proposed solutions. The authors in [128,129,130,131,132] did not evaluate performance and the capability of the solutions against DDoS attacks. In addition, these solutions just simply rely on the Ethereum platform without any extra working mechanism for detecting and mitigating DDoS attacks. Even without an attack, the performance of the systems under the Ethereum platform is worrying. This is because only by submitting normal transactions is it possible to cause a service disruption on the Ethereum platform. Furthermore, these solutions only prevent attacks in Ethereum based Blockchains. They cannot be readily used in other Blockchain-based platforms like Bitcoin, Ripple, etc.

#### 5.4.2. Solutions Based on the Ethereum Platform with Traffic Control (SEPTC)

In addition to the Ethereum platform, the solutions presented in [133,134,135] also pay attention to traffic control to mitigate DDoS attacks. Similar to the category of traffic control-based solutions (i.e., Section 5.3), in this classification, two types of strategies are used, which are the maximum rate of transactions [133] and whitelisting mechanism [134,135].

The solution proposed by [133] uses the maximum rate of transactions to prevent DDoS attacks. They take advantage of the gas limit attribute of the Ethereum to ensure that no more resources will be consumed if the gas consumption reaches the value of the threshold [133]. This mechanism has two benefits for preventing DDoS attacks. Firstly, because of the gas limitation, the server’s bandwidth can be protected from exhaustion even if all devices send data at the same time. Secondly, the malicious device itself needs to consume gas first to launch a DDoS attack. Once the limit is reached, the malicious traffic will be automatically terminated [133].

Solutions presented by [134,135] are based on the Ethereum platform and the whitelisting mechanism to mitigate DDoS attacks. Interestingly, Ref. [134] runs this system for more than 500 days with a large number of IoT devices and tested the effectiveness of mitigating DDoS attacks. The result of the experiment revealed that the latency under a DDoS attack is the same as that of normal traffic. In addition, CPU utilisation is only slightly more than normal circumstances. However, users used more bandwidth under the DDoS attack. It is noticed that Ref. [134] not only prevents DDoS attacks by applying whitelisting mechanism but also checks TLS handshakes to prevent layer 3 DDoS attacks.

Solutions in this category take advantage of the Ethereum platform and traffic control to mitigate DDoS attacks. However, combining both technologies also inherits their issues. For example, these solutions need to consider the limitation of the Ethereum platform’s performance on these solutions e.g., the performance can be affected because of the extra processing of traffic, high CPU utilisation, etc.

#### 5.4.3. Solutions Based on the Ethereum Platform with Authorization (SEPA)

These solutions combine the Ethereum platform with authorisation to prevent DDoS attacks [136,137,138,139]. The Ethereum platform itself does not filter incoming devices, leaving malicious devices free to use the platform. Refs. [136,137,139] authorise users by checking access tokens generated by a smart contract. Ref. [138] uses PUF technology to control IoT devices. This type of solution manages devices’ authorisation in addition to only relying on the platform. To some extent, this can help to prevent DDoS attacks. Interestingly, Ref. [136] presented that it is necessary to prevent DDoS attacks from triggering smart contracts. They proposed a mechanism in which each user must solve a cryptographic puzzle before a transaction.

Besides the prevention of DDoS attacks by payment of gas, these solutions can also prevent malicious users from accessing the systems. However, the issues of this type of solutions are that most of the solutions are in the initial stage, and no consideration is given to detecting and mitigating DDoS attacks. Once an attacker finds a way to bypass authentication and launch a DDoS attack, the system has no working mechanism to detect and mitigate the attack.

#### 5.4.4. Strengths and Weaknesses of the Ethereum Platform Based Solutions

Although it is convenient to use the Ethereum platform to deploy IoT devices, there are several weaknesses with the Ethereum platform-based solutions. To begin with, most of the solutions are in the initial stage, and they just simulate the implementation by using few nodes. The widespread use of the IoT requires the ability of scalability. However, with only a few nodes, it is hard to judge the availability and performance of these solutions. Furthermore, there are negative effects on the processing delays and costs on the Ethereum Blockchain when an attack occurs [139]. Even without an attack, submitting normal transactions might cause a service disruption on the Ethereum platform [140]. In addition, smart contracts need to be protected because they are susceptible to security failures. Attackers can make a particular smart contract operate incorrectly and thus cost money [140]. However, the only solution proposed in [135] considered DDoS attacks that trigger smart contracts. Finally, coupling with a third-party platform might lead to unpredictable issues [123], such as unpredictable transaction latencies, the time cost to update the Blockchain, the requirement of large storage size, etc. [140]. The summary of these three categories is shown in Table 6.

A complete list of the acronyms used in this research is presented in Table 7.

## 6. Future Research Directions

In this section, we present future research directions that can be explored further by other researchers. These future research directions are given below:

### 6.1. Protection of Smart Contracts

On the Ethereum platform, smart contracts are used to enforce specific rules to audit access policies to manage and control IoT devices. When defending against DDoS attacks, the systems rely on the security of Blockchain and smart contracts to reduce the impact. However, little attention is paid to the fact that smart contracts are themselves vulnerable to security issues and need to be protected. Once the smart contract is attacked, there will be economic losses and the specific smart contract will not work. Many popular Blockchain platforms use smart contracts, such as Ethereum, Fabric, etc. However, our survey results show that only Ref. [135] considered the protection of smart contracts. Therefore, one of the future research directions is to protect smart contracts.

### 6.2. Portability of Mitigation Solutions

Mitigation solutions for DDoS attacks should be portable and not simply dependent on one platform. The mechanisms should still protect the system when migration to other platforms is carried out. However, many solutions [128,129,130,131,133,134,135,136,137,138,139] are based on the Ethereum Platform and cannot be readily used on other blockchain based platforms. Therefore, another future research direction is to design and develop portable solutions to mitigate DDoS attacks.

### 6.3. Combination of Prevention, Detection and Reaction Mechanisms for DDoS Attacks

The three defence mechanisms against DDoS attacks are prevention, detection and reaction [4]. The defense mechanisms used by different solutions are mentioned in Table 3, Table 4, Table 5 and Table 6. Prevention is considered as the best strategy to mitigate DDoS attacks and most of the solutions [104,105,107,108,110,112,113,114,115,116] used this strategy. Prevention increases security but cannot completely stop DDoS attacks. By using detection methods, systems can detect DDoS attacks, and few solutions considered this mechanism [124,125,126,127,133,134,135]. Similarly, upon detection of DDoS attack, a reaction mechanism is used to stop the attack, to find the attacker, to recover from attack, etc. However, many solutions do not consider the reaction mechanism. Only Refs. [112,113,114,115,116,126,133,134,135] have considered the reaction mechanism. Therefore, future solutions should be proposed that take into account all three defense mechanisms against DDoS attacks.

### 6.4. Different Types of DDoS Attacks

Our survey results show that most solutions just mentioned that they are mitigating DDoS attacks in IoT-based networks without specifically mentioning the type of DDoS attack. Only few solutions [112,113,115,134,135] specifically mentioned the type of DDoS attack that they are mitigating. TCP SYN flood, UDP flood and ICMP flood are a few of the DDoS attacks that are considered by [112,113,115,134,135]. However, other DDoS attacks that can occur in IoT networks e.g., Hello flood, HTTP flood, DNS amplification, etc. are not considered by the existing solutions. Therefore, future researchers should propose Blockchain-based solutions to mitigate these DDoS attacks in IoT.

### 6.5. DDoS Attacks on IoT Based on Recently Known Botnets

In recent years, many sophisticated malware have been discovered by researchers that can make millions of IoT devices part of a botnet to carry out DDoS attacks. These malware include the famous Mirai Botnet which infected 15 million IoT devices with a flooding speed of 1 Tbps [141]. Other similar malware includes Reaper, Torii [142,143,144], etc. Hence, new schemes are essential to evaluate within the environment capable of replicating real-time conditions, both in terms of traffic scenarios and IoT infrastructure. This will also ensure the scalability of preventing DDoS attacks within the increased traffic demands and the heterogeneity of network structure.

Recall that the IoT ecosystems operate over a number of applications with different communication and security requirements (e.g., real-time delays intolerant applications such as health and disaster recovery applications or traffic control and smart home scenarios), which resulted in growing demands of intensive resource sharing and generic IoT infrastructures. Hence, new security solutions must be able to identify and prioritise malicious activities intended in different application scenarios. This will also enable the IoT infrastructure to be reconfigured at runtime. Furthermore, the routing protocols (including routing protocols for Mobile IoT [59]) for IoT need to be explored for their routing mechanisms and to search for opportunities in their routing mechanism to detect and mitigate DDoS attacks.

### 6.6. Large-Scale IoT Network and Scalability

The wide application of IoT determines its high scalability requirement. However, only two solutions [115,134] used relatively large-scale devices to verify the feasibility of their solution. For example, Ref. [115] conducted an experiment with 6000 IoT forwarding devices, and Ref. [134] used hundreds of devices. Simulations of using only a few nodes are far away from the actual scenario in IoT to evaluate the ability of any solution to mitigate DDoS attacks. Therefore, future research directions should include large-scale IoT networks.

As discussed earlier that the detection accuracy is high on the victim side, it is not robust enough to prevent the system because of massive DDoS attacks. Furthermore, it is highly possible that the intermediate network nodes are highly impacted because of the high-volume attacks generated at source IoT devices. Hence, first, it would be best to stop attacks at the sources; however, it is difficult to differentiate the traffic at sources because of minor traffic over the IoT devices. In addition, there should be centralised mechanisms deployed with various defense components i.e., prevention, detection and response, in order to deal with the DDoS flooding attacks.

In addition, there should be an authentication mechanism of IoT devices (specifically for Mobile IoT [59]) within the participating network so that the IoT devices could be made accountable for malicious activities generated at them. This also directs for introducing trusted infrastructures in order to enable trusted cooperation and collaboration among distributed components.

### 6.7. Compatibility of Solutions with Different IoT Application Domains

Considering the heterogeneity and usage of IoT devices in different industries, compatible and portable Blockchain-based solutions that mitigate DDoS attacks should be proposed in the future. However, our survey shows that all the solutions are used in specific situations with no proof that the mitigation mechanism is suitable for various industries in IoT. For example, solutions in [128] can only be used in firmware upgrade area; Refs. [108,120] are applied in smart homes; Refs. [107,136] are used in healthcare. Therefore, future research should be directed towards proposing solutions that can be used in multiple IoT application domains.

### 6.8. Comprehensive Experiments

Our survey results show that only four solutions [112,113,115,134] carried out experiments to determine the performance of their proposed solution. However, the experiments carried out by [112,113,115,134] are not comprehensive. This is because all of them do not consider multiple types of DDoS attacks on a large number of IoT devices. Moreover, experiments carried out in [112,113,115,134] fail to consider the prevention, detection and reaction ability of the solution; time is taken by solutions to detect and mitigate DDoS attacks, etc. Therefore, comprehensive experiments should be carried out in the future to properly gauge the effectiveness of the proposed solution.

### 6.9. Hybrid Platforms for Securing IoT Using Blockchain

The Blockchain is a powerful technology to secure the internet; however, the adoption of the Blockchain in IoT is still under development and is facing major challenges due to the limited capabilities of IoT devices in terms of resources, scalability and securing the overall network. A recent work [145] proposes a solution for protecting IoT devices from Mirai botnet attacks using Blockchain, suggesting dividing the network into different Autonomous Systems (AS) that monitor the communication activity within the network and determine whether a host is infected with malware. Hence, researchers are proposing the use of Blockchain in IoT using various technologies; we discuss a few challenges and possible research directions as follows:

#### 6.9.1. Edge Computing and Blockchain IoT

Recall that the IoT devices pose several challenges while integrating with the blockchain technology due to their limited computation and networking capabilities (as IoT devices use traditional ways for connecting to the internet). Then, the IoT devices cannot process Proof-of-Work consensus algorithms due to limited computational power and constrained battery lifetime. The edge devices also lack the authorisation and authentication capabilities and limited interoperability [146], which can be enabled by implementing blockchain technology. Although there are several research works e.g., reputation and trust integration model between IoT and fog/edge computing [147], a blockchain-based reputation model for each participating agents [148], the consortium blockchain proposal for efficient and secure knowledge trading in edge AI-enabled IoT [149], and lightweight blockchain framework using a dynamic trust algorithm in IIoT edge applications [150]. These proposals suggest the integration of IoT devices with edge computing while extending the capabilities of blockchain technology. However, a major challenge is to propose a hybrid infrastructure in order to integrate IoT and edge computing with the blockchain without publishing blockchain transactions in a centralised pool as is done in traditional blockchain networks.

#### 6.9.2. Software-Defined Internet of Things, Blockchain and Edge

As mentioned earlier, there are very few works that present a combined architecture including SDIoT [78,79,80,81,82] and SDIoT-Edge frameworks [83,84] suggested for effective management, increased scalability and interoperability. However, we note that (to the best of our knowledge) there are no works that study the heterogeneous service architecture that combines SDIoT-Edge with blockchain. This will enable a diverse combination of platforms, network topologies, protocols and various technologies, which will equip the IoT networks with increased security. This will pose several challenges in order to propose, operate, manage and secure applications that would operate on heterogeneous platforms. In addition, the security challenges can be addressed by providing network-based solutions and over the interface between the IoT networks combined with other technologies.

#### 6.9.3. High-Speed Cellular 5G/6G Networks

The high-speed 5G/6G networks have enabled seamless communication among different platforms, e.g., SDIoT, SDIoT-Edge, blockchain, etc., and have increasingly been deployed as a communication resource. Although this will create immense opportunities for the researchers to develop the standards and protocols in order to coexist the wireless SDN with the IoT networks [58]. It is noted that the security features of blockchain can be implemented in IoT edge devices by separating the control layer from the application layer that will help in implementing various features within the cellular network such as data authentication and protection. However, an energy-efficient resources provision e.g., in SDIoT-Edge and privacy are still challenging [151]. In addition, the network scalability will still be an issue because of a high volume of data exchange over IoT devices, in addition to managing synchronising protocols for data exchange [152,153].

## 7. Conclusions

In this survey, we reviewed how the IoT networks are vulnerable to the DDoS attacks by exploiting IoT devices to target legitimate services. We discuss the DDoS attack scenario, its effect on IoT network and connected services, the layer of impact, the integration of Blockchain in IoT and its potential use to address DDoS attacks; in addition, we further briefly discuss challenges of Blockchain implementation in IoT. We discuss various existing Blockchain-based solutions to mitigate the DDoS attacks in the IoT environment and further categorise them into four categories—Distributed Architecture-based solutions, Access Management-based solutions, Traffic Control-based solutions and the Ethereum Platform-based solutions. We analyse existing solutions under each category for their working principles, the DDoS attack mitigation (i.e., prevention, detection, reaction), along with their weaknesses and strengths. Various research directions are also proposed in this survey that will enable future researchers to propose better Blockchain-based solutions to mitigate DDoS attacks in IoT. This work limits its investigation to security issues related to the Network layer of IoT and does not classify existing solutions that address security issues related to IoT’s Application and Sensor layers. In addition, we did not discuss the solutions that address the privacy issues. Furthermore, this article limits its study to Blockchain-based solutions, and we plan to see how the DDoS security (along with privacy) issues were addressed using Machine Learning or other technologies.

## Figures and Tables

**Table 1:** Summary of literature works.

Referenced Works	Explanation of Blockchain	Security Issues in IoT	DDoS Attacks	Benefits and Challenges of Combining IoT and Blockchain	General Defense Mechanisms against DDoS Attacks	Usage of Blockchain in Security	Solutions for DDoS Attacks in IoT
[20]	Yes	Yes	No	Yes	No	No	No
[94]						Yes	
[92]				No		No	
[12]				Yes			
[95]						Yes	
[96]							
[90]						No	
[97]						Yes	
[5]						No	
[98]	No	No	Yes	No	Yes		
[4]							
[15]							
[99]							
[100]							
[102]		Yes	No				Yes
[103]		No					
[101]		Yes					No

**Table 2:** Distribution of research articles with various publishers.

Resources	IEEE Xplore	Elsevier	ScienceDirect	Springer	ProQuest	ACM	
Conference	32	0	238	159	429	52	
Journals	7	19		156		25	
Books	24	200	22	345	2132	0	
Early Access Articles	1	0	0	0	0	0	
Magazines	1	0	0	0	20	0	
Webpages	0	0	0	0	16	0	
Connect	0	1	0	0	0	0	
Survey Articiles	0	0	76	0	0	0	
Discussion	0	0	1	0	0	0	
Editorials	0	0	1	0	0	0	
Dissertations & Thesis	0	0	0	0	20	0	
Newsletter	0	0	0	0	0	6	
Reports	0	0	0	0	0	0	
							Total
Total	65	220	338	660	2617	83	3983
Excluded (Books, etc.)	26	201	100	345	2188	6	2866
Included (Conf. Jour.)	39	19	238	315	429	77	1117
The excluded 2866 articles were Books, Reports, etc. Following is the breakdown of resultant 1117 research articles:							
Blockchain-based Solutions = 34; Survey Articles = 12; Supporting Articles = 103; Remaining = 1117 − 149 = 968							
Finally, 968 research articles were excluded from our repository that were not classified as Blockchain-based Solutions.							

**Table 3:** Summary of distributed architecture based solutions.

Solutions	Working Principle	DDoS Attack Mitigation			Weaknesses	Strengths
		Prevention	Detection	Reaction		
[104]	The systems are decentralised, and all the nodes share the ledger with redundancy data storage.	Yes	No	No	Nodes not under attack will be under heavy load.	Utilise natural feature of Blockchain to mitigate DDoS attacks.
[105]	Use the distributed structure of Blockchain to mitigate DDoS attacks.	Yes	No	No	Specific node under attack can not work.	When DDoS attacks occur, the whole system continues to work.
[106]	Uses the collaborative DDoS detection scheme utilising Blockchain and lightweight agents in IoT.	Yes	Yes	Yes	Details over the consensus algorithm on agents are missing, since these algorithms are supposed to be installed over limited resources hardware.	The use of lightweight agents exchange outbound traffic information to identify possible victims of DDoS attacks and is governed by a Blockchain smart contract, which ensures the integrity of both the procedure and exchanged information.

**Table 4:** Summary of access management based solutions.

Solutions	Working Principle	DDoS Attack Mitigation			Weaknesses	Strengths
		Prevention	Detection	Reaction		
PKAM [107,108,109]	Based on public key to manage access. Reject requests if the requester’s public key is not registered or unavailable.	Yes	No	No	Cannot prevent DDoS attacks if attackers use multiple public keys.	Prevent DDoS attacks by limiting unauthorised access.
PUFAM [7,110]	Use PUF to verify the authenticity of the IoT devices. All tampered, fake and cloned devices will be detected, which can prevent devices from becoming part of a botnet.	Yes	No	No	No experiments to prove the robustness against DDoS attacks.	It is a lightweight access management solution which is suitable for an IoT environment.

**Table 5:** Summary of traffic control based solutions.

Solutions	Working Principle	DDoS Attack Mitigation			Weaknesses	Strengths
		Prevention	Detection	Reaction		
SDNTCB [112,113,114,115,116,117,118]	Combine SDN and Blockchain to monitor traffic to detect DDoS attacks.	Yes	Yes	Yes	Delay caused by processing of traffic is not calculated.	Sound mechanism to mitigate DDoS attacks.
TCMRT [120,121]	If the threshold of maximum transaction rate is exceeded, the node manager updates to prevent the node from continuously sending transactions to the target nodes.	Yes	Yes	Yes	Can create too much traffic in the network.	A lightweight mechanism to mitigate DDoS attacks.
TCVT [123]	Verify outgoing transactions to prevent nodes from becoming part of botnets.	Yes	No	No	Lack of protection of smart contract.	Does not utilise additional resources.
TCWM [124,125,126,127]	A whitelisting mechanism is used to prevent DDoS attacks by filtering and eliminating malicious traffic.	Yes	Yes	Only [126]	It is possible that illegal traffic complies with validation rules but perform harmful actions.	It is very quick to verify the access traffic and filter the unwanted traffic.

**Table 6:** Summary of the Ethereum Platform based solutions.

Solutions	Working Principle	DDoS Attack Mitigation			Weaknesses	Strengths
		Prevention	Detection	Reaction		
SSEP [128,129,130,131,132]	Prevent attackers from sending too many service requests because of payment of transactions.	Yes	No	No	No mechanism for detecting and mitigating DDoS attacks.	Use existing Ethereum platform to prevent DDoS attacks.
SEPTC [133,134,135]	Combines the Ethereum platform and traffic control to mitigate DDoS attacks.	Yes	Yes	Yes	Decrease in performance because of extra processing of data.	Use maximum rate of transactions and white listing mechanisms.
SEPA [136,137,138,139]	Combines the Ethereum platform with authorisation to prevent DDoS attacks.	Yes	No	No	Do not consider detection and mitigation of DDoS attacks.	Can also prevent malicious users from accessing the systems.

**Table 7:** List of acronyms and their explanation.

Acronyms	Explanation
DDoS	Distributed Denial of Service
IoT	Internet of Things
IIoT	Industrial Internet of Things
IoA	Internet of Anything
IoE	Internet of Everything
SIoT	Social Internet of Things
WoT	Web of Things
IoMT	Internet of Medical Things
SDIoT	Software-Defined Internet of Things
SDIoT-Edge	Software-Defined Internet of Things and Edge
SDN	Software Defined Networking
PoS	Proof of Stake
BFT	Byzantine Fault Tolerance
PoET	Proof of Elapsed Time
ECDSA	Elliptic Curve Digital Signature Algorithm
HTTP	Hyper Text Transfer Protocol
VoIP	Voice over Internet Protocol
SHA	Secure Hash Algorithm
6LoWPAN	IPv6 over Low-Power Wireless Personal Area Networks
PKAM	Public Key based Access Management
PUF	Physically Unclonable Function based Access Management
PUFAM	PUF based Access Management
SDNTCB	SDN based Traffic Control via Blockchain
TCMRT	Traffic Control based on the Maximum Rate of Transactions
TCVT	Traffic Control based on Verification of Transactions
TCWM	Traffic Control based on Whitelisting Mechanism
TCP	Transmission Control Protocol
SYN	Synchronise
DNS	Domain Name System
CoAP	Constrained Application Protocol
PoW	Proof of Work
DPoS	Delegated-Proof-of-Stake
PoET	Proof of Elapsed Time
PoL	Proof of Luck
PoSp	Proof of Space
PBFT	Practical Byzantine Fault Tolerance
ePBFT	Excellent Practical Byzantine Fault Tolerance
PoL	Proof of Luck
CA	Certificate Authority
PK	Public Key
SC	Smart Contracts
BTC	Bitcoin
ICMP	Internet Control Message Protocol
SSEP	Solutions Simply based on Ethereum Platform
SEPTC	Ethereum Platform with Traffic Control
SEPA	Solutions based on the Ethereum Platform with Authorization
TLS	Transport Layer Security
CPU	Central Processing Unit
SSL	Secure Sockets Layer
IP	Internet Protocol
UDP	User Datagram Protocol
ICT	Information and Communication Technologies
ITU	International Telecommunication Union
RFID	Radio Frequency Identification

## Data Availability

There is no data associated with this article.
